# Medical Items

**Published:** 1896-12

**Authors:** 


### MEDICAL ITEMS.

Dr. W. C. Humphries, Acworth, Ga., writes that he has a first-class Beck microscope to sell. Any one wishing a microsope at a reasonable price would do well to write to Dr. Humphries. H.

---

The Managing Editor, Dr. Grandy, has returned from New York, where he has been pursuing studies in the Eye, Ear and Throat. Hereafter he will confine his practice to diseases of these organs.

---

"Doctor," said the patient, "I believe there is something wrong with my stomach." "Not a bit," replied the doctor promptly. "God made your stomach, and he knows how to make them. There's something wrong with the stuff you put in it, may be, and something wrong in the way you stuff it in and stamp it down, but your stomach is all right." And immediately the patient discharged him.—*Ex.*

---

The editors of *Mathews' Medical Quarterly* announce that with the January issue of that publication its name will be changed to *Mathews' Quarterly Journal of Rectal and Gastro-Intestinal Diseases.* This is a change which has been deemed necessary for some time, as it is essential that the title of a medical journal should convey to the reader an idea of its contents, and this has not been the case with its name from the beginning. There will be no change in the policy of the journal in the least. As it will continue to be the only English publication devoted to diseases and surgery of the rectum and gastro-intestinal tract, the articles which will appear in it will be limited to these subjects. The journal will continue to be edited by Drs. J. M. Mathews and Henry E. Tuley, and published in Louisville, Ky.

THE AMERICAN ASSOCIATION OF OBSTETRICIANS AND GYNECOLOGISTS, at its ninth annual meeting, held in Richmond, Va., elected the following-named officers for the ensuing year, namely: President, James F. W. Ross, M.D., Toronto; Vice-Presidents, George Ben Johnson, M.D., Richmond, and John C. Sexton, M.D., Rushville, Ind.; Secretary, William Warren Potter, M.D., Buffalo; Treasurer, Xavier O. Werder, M.D., Pittsburg. Executive Council: Charles A. L. Reed, M.D., Cincinnati; Lewis S. McMurtry, M.D., Louisville; A. Vandeveer, M.D., Albany; J. Henry Carstens, M.D., Detroit, and William E. B. Davis, M.D., Birmingham.

The next annual meeting was appointed to be held at the Cataract House, Niagara Falls, N. Y., Tuesday, Wednesday, Thursday, and Friday, August 17, 18, 19, and 20, 1897.

---

DR. JOHN B. HAMILTON, editor of the *Journal of the American Medical Association*, has resigned from the U. S. Marine Hospital Service, of which he was formerly Surgeon General, rather than submit to a transfer from Chicago to San Francisco, which must have hampered him in his editorial work, if it would not have quite prevented it.

The American Medical Association is to be congratulated upon its good fortune in retaining Dr. Hamilton in active charge of the *Journal*, which he has brought from comparative obscurity to a position second to that of no medical publication in the country, and which shows in each issue an imprint of his personality that would be missed by all who esteem the combination of broad scholarship, professional and literary acumen and executive ability—qualities so desirable in an editor, and possessed by Dr. Hamilton in an unusual degree.—*Ex.*

